# Longevity record of arctic skua (*Stercorarius parasiticus*)

**DOI:** 10.1002/ece3.6875

**Published:** 2020-10-06

**Authors:** Elina Mäntylä, Kari Mäntylä, Jukka Nuotio, Kimmo Nuotio, Matti Sillanpää

**Affiliations:** ^1^ Section of Ecology Department of Biology University of Turku Turku Finland; ^2^ Institute of Entomology Biology Centre of the Czech Academy of Sciences České Budějovice Czech Republic; ^3^ Faculty of Sciences University of South Bohemia České Budějovice Czech Republic; ^4^ Pori Ornithological Society Pori Finland; ^5^ Environmental Agency Pori Finland

**Keywords:** arctic skua, breeding, geolocator, longevity, migration, seabird

## Abstract

The arctic skua (*Stercorarius parasiticus*) is one of the most long‐lived bird species. In 2010, we captured in Finland an adult, female arctic skua which had been ringed as a nestling in 1987. We tagged it also with a color ring. The bird has last been seen in July 2020 at the age of 33 years, making it most likely the oldest known arctic skua of the world. In 2010–2011 the bird carried a light‐level measuring geolocator, the data of which revealed that the bird had spent the nonbreeding season in the Canary Current area on the west coast of Africa. Breeding populations of arctic skuas have declined recently especially in British Isles, thus it is useful to get longevity data of this species with a high breeding site fidelity.

## Graphical abstract

Arctic skuas (Stercorarius parasiticus) are one of the most long‐lived bird species. In Satakunta, Finland is breeding a female that was ringed as a nestling in 1987, meaning that it is now 33 years old. In 2010–2011 the bird carried a light‐level measuring geolocator, so we know that it spends the non‐breeding season in the Canary Current area by the west coast of Africa.

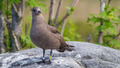

## 1 INTRODUCTION

Bird ringing has revealed that many bird species, especially seabirds, can live over 30 years in nature (Euring, 2020). For example, the oldest manx shearwater (*Puffinus puffinus*) has been over 50 years old, common guillemot (*Uria aalge*) almost 46 years, fulmar (*Fulmarus glacialis*) over 43 years old, and great skua (*Catharacta skua*) and lesser black‐backed gull (*Larus fuscus*) over 34 years old. The oldest known ringed bird of the world is the 69‐year‐old Laysan albatross (*Phoebastria immutabilis*) named Wisdom, ringed in 1956. In February 2019, she hatched again a chick.

One species that is known to live long is the arctic skua (*Stercorarius parasiticus*). The species has a circumpolar breeding distribution by the northern seas (Furness, 1987; Olsen & Larsson, 1997). Most of the individuals spend the nonbreeding season in the southern hemisphere (Furness, 1987; Olsen & Larsson, 1997). Depending on the location, the diet of the arctic skuas can vary from small rodents and fish to kleptoparasitic stealing of food from other birds, such as terns (*Sterna* sp.) and gulls (*Larus* sp.) (Grant, 1971; Taylor, 1979). Arctic skuas form life‐long pair bonds, being monogamous (Olsen & Larsson, 1997), and the birds normally lay two eggs (O’Donald, 2009).

Between 1965 and 1974, two Finnish bird ringers Pentti Forstén and Arvo Tuominen, ringed over 1,000 arctic skuas in western Finland. One bird that was ringed as a nestling in 1971 was found having died in 2002 in Sweden. It was then the oldest known arctic skua of the world, with the age of 31 years and 12 days (Euring, 2020). In 2017 a dead arctic skua was found in Finland and it had been ringed as nestling in 1989, meaning that it had most likely reached the age of 28 years. One arctic skua was ringed in Slettnes, Norway in 1991 and controlled at the same place in 2018 when it was 27 years old (Husebø & Mjøs, 2020). The oldest arctic skua in Iceland has been 26 years old (Icelandic Bird Ringing Scheme , 2020), in British Isles 25 years 10 months (Mead & Clark, 1991), and in Faroe Islands 20 years 11 months (The Faroese Bird Migration Atlas, 2014). The legal persecution of the species in Faroe Islands may explain the shorter longevity there.

## 2 Methods

**Figure 1:**
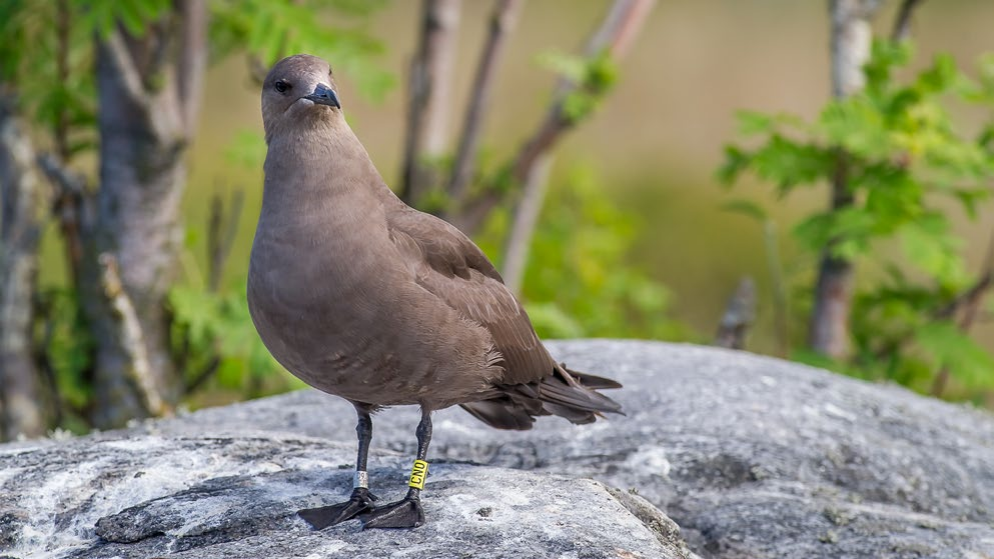
The arctic skua CN0. Photo taken by Matti Sillanpää

We started our arctic skua project in Satakunta, on the west coast of Finland, in 2010. The goal of the project was to record the migration routes and the nonbreeding areas of the arctic skuas by tagging adult individuals with light‐level measuring geolocators. The coasts of Finland are filled with rocky skerries and small islands, and arctic skuas breed on some of them as individual pairs and not as colonies as is typical in most of the other breeding areas of the species (Andersson & Götmark, 1980). On 15th June 2010 K. Nuotio and M. Sillanpää went to a ridge island known as a long‐time breeding territory of the arctic skua. They noticed that the female of the pair carried a steel ring and managed to capture it to control the ring. The arctic skua ST‐109.326 was also tagged with a color ring CN0 and a geolocator (MK15, British Antarctic Survey). The Finnish Bird Ringing Centre informed that the bird had been ringed as a nestling on 1st July 1987 by Kari Mäntylä, just over 8 km from the current location. In 2010, CN0 was almost 23 years old (Figure 1).

## 3 RESULTS AND DISCUSSION

**Figure 2:**
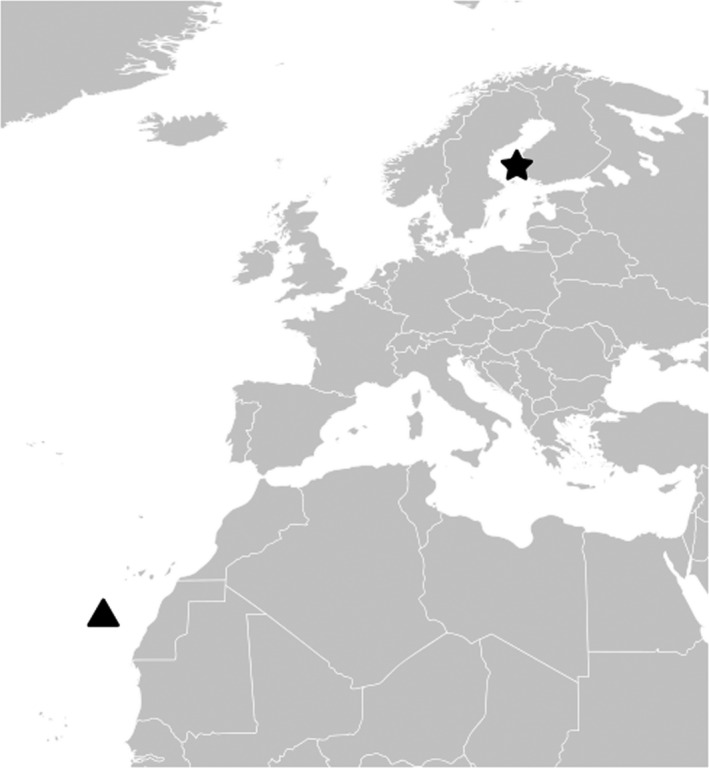
The breeding location of the arctic skua CN0 (star) and the nonbreeding season area (triangle). The nonbreeding site location is based on the geolocator data (van Bemmelen et al., 2019)

In 2011 we managed to recapture CN0 to get the geolocator data. The analysis of the data revealed that CN0 had flown to the western coast of Africa and the Canary Current for the nonbreeding season (van Bemmelen et al., 2019; Figure 2). CN0 and other Finnish arctic skuas with geolocators are part of a larger European research project led by Rob van Bemmelen. After 2011 we have been able to follow CN0 at the breeding site yearly thanks to the color ring. The bird has always returned to breed on the same island and often has had one or two offspring (Table 1). Kari Mäntylä has ringed the arctic skua nestlings on that same island almost every year until 2010 and says that in several cases he has seen one of the parents carry a steel ring, at least from the mid‐1990s on. So, it may well be possible that CN0 has bred on the same island all her adult life, or at least a great part of it. Breeding site fidelity is typical behavior for arctic skuas (Olsen & Larsson, 1997). With years our interest in the longevity of the arctic skuas in general and of CN0 in particular has grown stronger.

**Table 1:** Number of nestlings ringed on the breeding island of CN0. On that island has always been breeding only one pair of arctic skuas each year. Kari Mäntylä ringed the nestlings until 2010, and since 2011 Kimmo Nuotio and Matti Sillanpää have ringed the nestlings. From 2008 on the nestlings have also got a color ring. Most likely CN0 has been breeding on that island at least from 1995

Year	Nestlings
1989	1
1990	1
1991	–
1992	–
1993	1
1994	–
1995	1
1996	1
1997	–
1998	1
1999	–
2000	–
2001	–
2002	–
2003	1
2004	–
2005	–
2006	1
2007	–
2008	1
2009	–
2010	2
2011	2
2012	–
2013	1
2014	–
2015	1
2016	2
2017	1
2018	–
2019	1
2020	2

As can be seen from Table 1 there were several years when no nestlings were ringed. Since arctic skuas (and other seabirds) in Satakunta breed on small, rocky islands the weather needs to be good for a successful landing there when visiting to ring the nestlings. And there is only a limited time you can stay on an island (max. 1 hr), so that the small nestlings are not harmed. Therefore, in some years there probably have been nestlings but they were not found and ringed. As each island is usually visited only 1–2 times during each breeding season, we do not have any information of the survival of the offspring. But it is still very likely that CN0 has had several fledglings during the years. At least in 2010, we know that its offspring survived until autumn migration.

On 18th July 2018 K. Nuotio and M. Sillanpää once again travelled to the breeding island of CN0. If it were seen, it would be now the oldest arctic skua of the world. And they saw it, 31 years and 19 days after it had been ringed as a nestling (Nuotio & Sillanpää, 2019). CN0 returned to breed also in 2019 and 2020, and it has been last seen on 16th July 2020 when it was at least 33 years and 15 days old.

Many arctic skuas fly to southern Atlantic for the nonbreeding season, but CN0 did not cross the equator during the nonbreeding season. Based on recent knowledge, individual arctic skuas almost always migrate to the same nonbreeding area each year (van Bemmelen et al., 2019), and that Canary Current area is one used by arctic skuas breeding in northern Europe (van Bemmelen et al., 2019). The shortest distance between the breeding place and nonbreeding season area of CN0 is 6,000 km. Arctic skuas start to breed at the age of 3–5 years (O’Donald, 2009). So, the minimum total migration distance of CN0, if it started to breed as 5‐year‐old, is 28 × 2 × 6,000 km = 336,000 km. In reality, the distance it has covered during all those years must be much bigger. Even if the migration distance was shorter than for some other arctic skuas, it would need to fly every day for foraging and other daily movements.

It is remarkable that we have managed to gather so much information of this one bird individual, and with only few methods. Regular yearly visits to the breeding island to ring the nestlings, capturing adults to check the metal rings and add the new color rings to get more easily resightings. Arctic skuas spend usually their nonbreeding time outside areas with human activity, so the best way to get information of migration and nonbreeding areas is with geolocators (or other trackers). One reason for the longevity of arctic skuas could be also the fact that they spend most of their time at the open sea, and do not fish themselves, reducing the possibility to become bycatch of fishing vessels. There is very little data of annual mortality of arctic skuas, but in Fair Isle the adult mortality in 1948–78 was 19.04% (O’Donald, 2009).

Long‐term monitoring and ringing of long‐lived bird species gives information of not only that bird individual or its species, but also possible changes in its breeding and nonbreeding environments.

## 4 DATA ACCESSIBILITY STATEMENT

The data are published in the article.

## CONFLICT OF INTEREST

None declared.

## AUTHOR CONTRIBUTION

**Elina Mäntylä:** Conceptualization (equal); Data curation (equal); Formal analysis (equal); Funding acquisition (lead); Investigation (equal); Methodology (equal); Project administration (equal); Resources (equal); Software (equal); Supervision (equal); Validation (equal); Visualization (equal); Writing‐original draft (lead); Writing‐review & editing (lead). **Kari Mäntylä:** Conceptualization (equal); Data curation (equal); Formal analysis (equal); Funding acquisition (equal); Investigation (equal); Methodology (equal); Project administration (equal); Resources (equal); Software (supporting); Supervision (equal); Validation (equal); Visualization (equal); Writing‐original draft (supporting); Writing‐review & editing (supporting). **Jukka Nuotio:** Conceptualization (supporting); Data curation (supporting); Formal analysis (supporting); Funding acquisition (equal); Investigation (equal); Methodology (equal); Project administration (supporting); Resources (equal); Software (supporting); Supervision (supporting); Validation (supporting); Visualization (supporting); Writing‐original draft (supporting); Writing‐review & editing (supporting). **Kimmo Nuotio:** Conceptualization (equal); Data curation (equal); Formal analysis (equal); Funding acquisition (equal); Investigation (lead); Methodology (equal); Project administration (equal); Resources (equal); Software (equal); Supervision (equal); Validation (equal); Visualization (equal); Writing‐original draft (supporting); Writing‐review & editing (equal). **Matti Sillanpää:** Conceptualization (equal); Data curation (equal); Formal analysis (equal); Funding acquisition (equal); Investigation (lead); Methodology (equal); Project administration (equal); Resources (equal); Software (equal); Supervision (equal); Validation (equal); Visualization (equal); Writing‐original draft (supporting); Writing‐review & editing (equal).
